# *Fructus Amomi* extract attenuates nasal inflammation by restoring Th1/Th2 balance and down-regulation of NF-κB phosphorylation in OVA-induced allergic rhinitis

**DOI:** 10.1042/BSR20212681

**Published:** 2022-03-17

**Authors:** Yanjing Fan, Thi Van Nguyen, Chun Hua Piao, Hee Soon Shin, Chang Ho Song, Ok Hee Chai

**Affiliations:** 1 School of Medicine, Liaocheng University, Liaocheng, Shandong, People’s Republic of China; 2 Department of Anatomy, Jeonbuk National University Medical School, Jeonju, Jeonbuk 54896, Republic of Korea; 3 Department of Pulmonary and Critical Care Medicine, Yantai Yuhuangding Hospital, Yantai 264000, People’s Republic of China; 4 Division of Food Functionality Research, Korea Food Research Institute, 245 Nongsaengmyeong-ro, Iseo-myeon, Wanju-gun, Jeollabuk-do 55365, Republic of Korea; 5 Department of Food Biotechnology, University of Science and Technology, Daejeon 34113, Republic of Korea; 6 Institute for Medical Sciences, Jeonbuk National University, Jeonju 54896, Republic of Korea

**Keywords:** allergic rhinitis, Fructus Amomi, mast cell degranulation, NF-κB phosphorylation, Th1/Th2 balance

## Abstract

*Fructus Amomi* Cardamomi (FA) is the mature fruit of *Amomum villosum* Lour (family *Zingiberaceae*) and is commonly used in Chinese traditional medicine to treat various gastrointestinal disorders. FA’s possible benefits as an allergic rhinitis (AR) treatment, however, have not been examined. We used an ovalbumin (OVA)-induced AR mouse model to identify any anti-allergic effects associated with the administration of 200 mg/kg FA or dexamethasone (Dex) 2.5 mg/kg by oral administration. The results of our testing confirm that FA ameliorated nasal symptoms and alleviated nasal epithelium swelling, reduced the goblet cell hyperplasia and eosinophil cell infiltration in the nasal epithelium, and inhibited lung tissue inflammation and Dex as well. Significantly decreased Th2 cytokine (interleukin (IL)-1β, IL-4, and IL-5) expression, and a correspondingly significant increase in Th1 cytokine (IL-12, interferon (IFN)-γ) production, was observed in nasal lavage fluid (NALF) taken from mice that received FA or Dex treatment. FA also reduced the presence of OVA-specific immunoglobulin (Ig) E, OVA-specific IgG_1_, and histamine levels in serum, and inhibited mast cell degranulation *in vitro*. In addition, these effects were involved with the reduction in NF-κB phosphorylation. These results suggest that FA restores Th1/Th2 balance and inhibits NF-κB phosphorylation and mast cell degranulation, thereby achieving a notable anti-inflammatory effect. Accordingly, it has the potential to be used as an efficacious therapeutic treatment for AR.

## Introduction

Allergic rhinitis (AR) is a common inflammatory disorder of the nasal airways that is characterized by the appearance of one or more symptoms, including nasal congestion, rhinorrhea, sneezing, nasal itching [1]. It frequently coincides with other allergic inflammatory diseases such as asthma, rhinosinusitis, allergic conjunctivitis, and adenoid hypertrophy [2]. AR symptoms appear after exposure to allergens, and are immunoglobulin (Ig) E-mediated hypersensitivity reactions [3]. Typical allergens that trigger AR include dust, pollen, spores, and pet dander. T helper 1 (Th1) and Th2 cells both play a role in the pathogenesis of allergic inflammatory diseases like asthma or AR [4]. The IgE-mediated response characterized by a Th2-immunologic pattern with mast cells releases several mediators, chemokines, and cytokines that recruit eosinophils and other components, that in turn contribute to inflammation [5]. Th2 cell is a key factor in the development of AR, and Th2 lymphocytes may be a viable therapeutic target to treat the condition [5]. Mast cells are allergy cells responsible for immediate allergic reactions, triggering allergic symptoms by releasing its mediators. Histamine is a typical mast cell mediator that may induce allergic symptoms such as itching, sneezing, or a running nose [6]. The critical role of NF-κB signaling pathway in inflammatory progression was well known to induce the expression of several pro-inflammatory genes in innate immune cell, regulate the differentiation of inflammatory T-helper cells, and participate in inflammasome regulation [7].

A number of pharmacologic options exist to treat AR, including anti-histamines, corticosteroids, anti-cholinergic agents, leukotriene inhibitors, and immunotherapy. By far, the most common treatment method is intranasal corticosteroids [8]. The long-term use of steroids, however, is associated with adverse effects, including intraocular pressure elevation [9], nasal dryness, and disruptions to taste and smell [10]. There is a need, therefore, for new, safer, and more economical alternative therapeutic agents to treat allergic disorders.

Natural products are widely used in the treatment of various chronic human pathological conditions. *Fructus amomi* Cardamomi (FA)—the ripe fruit of *Amomum villosum* Lour of the family *Zingiberaceae*—has been shown to attenuate mast cell activity and reduce TNF-α and interleukin (IL)-6 cytokine expression [11]. Its primary active ingredient, essential oil, is often used to drive away damp, improve appetite, warm the spleen, stop diarrhea, prevent spontaneous abortion, and have positive effect in gastric cancer [12]. To date, the anti-allergic effect of FA on rhinitis has not been clear. Therefore, the present study aims to investigate the effect of FA on an OVA-induced rhinitis mouse model.

## Materials and methods

### Animals

Seven-week-old male BALB/c male mice with bodyweight approximately 21–23 g and rats 8-week-old male approximately 250 g were provided by Damool Science (Daejeon, Korea). These mice and rats were maintained in an air-conditioned, free OVA room (23–25°C) with a 12-h light/dark cycle. All animal experiments were performed at Department of Anatomy Jeonbuk National University Medical School and approved by the Institutional Animal Care and Use Committee of Jeonbuk National University (JBNU 2021-0115, 27 July 2021).

### FA ethanol extract preparation

The FA used in the present study was obtained from Kyeong-Dong Oriental Pharmacy Market (Seoul, Korea) and identified by Professor Y. Bu, Department of Herbal Pharmacology, Kyung Hee University. FA ethanol extract (KFRI-SL-2119, FA) was prepared by the Korea Food Research Institute (Jeollabuk-do, Korea). The FA was extracted twice in 95% ethanol reflux, and the product was dried by a rotary evaporator. The dried FA powder was kept at 4°C until it was needed, and dissolved in saline prior to its use.

### Sensitization and treatment

**Figure 1:**
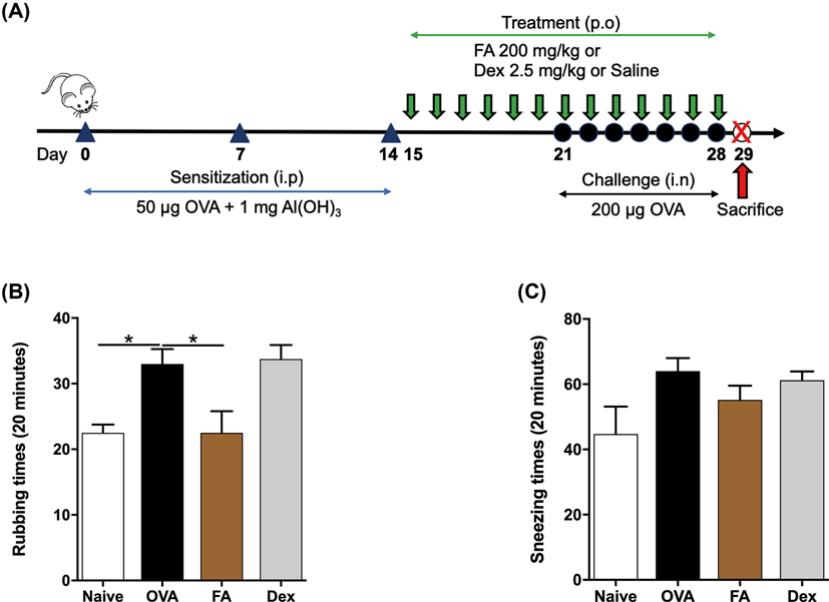
Effect of FA on nasal symptoms in the murine AR model (**A**) Animal protocol. Mice were sensitized on days 0, 7, and 14; challenged between days 21 and 28. Mice in the OVA group were sensitized and challenged by OVA. Mice in the FA or Dex treatment groups were sensitized and challenged by OVA and administered 200 mg/kg FA or 2.5 mg/kg Dex between days 15 and 28 once a day by oral gavage (200 μl). (**B**) Rubbing and (**C**) sneezing scores. The values represent the mean ± S.E.M (*n*=6/group). Significant differences at *P<0.05 are compared with each group.

BALB/c mice aged 6 weeks were divided into four groups (*n*=6) at random: Group 1 (Naive group); Group 2 (Ovalbumin (OVA) + saline group); Group 3 (OVA + FA 200 mg/kg group); Group 4 (OVA + dexamethasone (Dex)). To develop an AR murine model, mice were injected intraperitoneally with 50 μg OVA (Sigma, St. Louis, MO, U.S.A.) and 1 mg Imject Alum (Pierce, Rockford, IL, U.S.A.), which induced systemic sensitization in all but Group 1 (Figure 1). Between days 15 and 28, FA and Dex group mice received 200 μl FA (200 mg/kg) or Dex (2.5 mg/kg) by oral gavage, while mice in the OVA groups received the same volume of saline. Between days 21 and 28, the mice in the OVA, FA, and Dex groups were intranasally challenged with 20 μl of 10 mg/ml OVA solution injected into both nasal cavities. Mice were lightly anesthetized by inhaling ether (Cat. 071118, Samchun, Korea) and killed by cervical dislocation on day 28, 24 h after the last OVA challenge [13]. The mice were restrained on a cover of the mice cage, the tail was grasped with one hand. The back of the neck was tightened at the base of the skull by a sturdy stick-type pen. To produce the dislocation, quickly pushed forward and down with the hand while pulling backward with the hand holding the tail base. When the spinal cord was severed, a 2–4 mm space was palpable between the occipital condyles and the first cervical vertebra and there was no heartbeat. The rats used for rat peritoneal mast cell (RPMC) experiment were also killed by the same process as the mice.

### Measurement of nasal symptoms

Nasal symptoms, including rubbing and sneezing, occurring over a 20-min period were recorded, then counted by blind observers on the last day on which the mice were challenged with OVA.

### Serum and nasal lavage fluid collection

Mice were anesthetized with diethyl ether before scarification. Blood samples were harvested then centrifuged at 10000 rpm for 4°C to obtain serum. Nasal lavage fluid (NALF) was collected via an 18-gauge catheter. The trachea was partially resected, a catheter was inserted from the trachea and moved up and forward to the nasopharynx, and the NALF was harvested after 1-ml saline was gently perfused through the nasal passages. The NALF was centrifuged at 10000 rpm for 10 min at 4°C. The supernatant was kept at –80°C until for further analysis. The pellet was used to identify differentiated cells in the NALF by a cytospin device (Centrifuge 5403, Eppendorf, Hamburg, Germany) at 1000 rpm for 10 min [13,14].

### Histopathological analysis

Mice heads were fixed in 10% formalin for approximately 3 days at room temperature, then decalcified over 10 days in Calci Clear-Rapid (National Diagnostics, Atlanta, GA, U.S.A.), which contained hydrochloric acid and EDTA. The heads were continuously dehydrated by gradually increasing the concentration of ethanol and xylene and then embedded in paraffin. Samples were sectioned in 4.5-μm segments. The head sections were stained with Hematoxylin–Eosin (H&E) to evaluate overall inflammation. Periodic Acid–Schiff (PAS), Giemsa, and Toluidine Blue stain method was used to detect goblet cells, eosinophils, and mast cells.

Histopathologic analysis of the lungs was performed through these same procedures (absent decalcification). The paraffin section slides of the lung tissues were stained by H&E to observe general changes to morphology [15].

### Measurement of cytokine levels in NALF and Ig levels in serum

The levels of Th2 cytokines IL-4 and IL-5 in NALF, Th1 cytokines interferon (IFN)-γ and IL-12 in NALF, as well as OVA-specific IgE, OVA-specific IgG, and histamine in serum, were measured by enzyme-linked immunosorbent assay (ELISA) kits (BD Biosciences, San Diego, CA, U.S.A. and R&D Systems Inc., Minneapolis, MN, U.S.A.) consistent with the manufacturers instructions.

### Determination of NF-κB signaling pathway-related proteins by Western blot

The appropriate amount of lung tissue was weighed, and protein was extracted by RIPA buffer and a phosphatase inhibitor (100:1). The supernatant was centrifuged (4°C, 5000 rpm, 10 min), and the protein concentration was measured by BSA standard with Bradford dye (Bio-Rad Laboratories, Inc, U.S.A.). The samples were loaded on SDS/PAGE then transferred to a PVDF membrane, and block with 5% skim milk for approximately 1 h. The membrane was incubated with primary antibody overnight at 4°C by an orbital shaker. Then the membrane was washed with TBST then incubated with secondary antibody for 1 h at room temperature. The blot was detected by a detection solution.

### RPMC degranulation

RPMCs were harvested as described previously. In brief, rats were anesthetized by ether then injected with 10 ml of saline into the peritoneal cavity. The peritoneal cavity was opened, 50 ml saline was added, then the cavity was massaged gently (without touching the liver). The fluid was aspirated to another tube using a Pasteur pipette, then centrifuged at 1000 rpm for 10 min at 4°C. The supernatant was discarded, and the RPMCs were resuspended in HEPES (1 × 10^6^ cells/ml). Two-hundred microliters of RPMCs were pretreated with 25 µl saline (control group) or FA (0.01, 0.1, 1 mg/ml) for approximately 10 min at 37°C, then stimulated with 25 µl compound 48/80 (C48/80, Sigma, Cat. C2313) or saline for 15 min. After incubation, the mast cell degranulation rate (the degranulated mast cells/total mast cells × 100) was calculated based on observations made through a microscope [16].

### Statistical analysis

The statistical analysis was performed with GraphPad Prism software (version 5.0) (La Jolla, CA, U.S.A.). The data outputs were expressed as means ± SEM and analyzed by one-way ANOVA followed by Tukey’s test. Statistical significance was considered at *P*<0.05.

## Results

### Effect of FA on nasal symptoms in the murine AR model

To investigate FA’s possible anti-allergic effect, mice behaviors were recorded over an approximate 20-min period after OVA challenge on day 27. The frequency of nasal rubbing, and sneezing was counted. The OVA-induced AR mice group manifested significantly more nasal rubbing and sneezing than the Naive group. The incidence of nasal symptoms was reduced by treatment with FA. The mice in the Dex group also manifested fewer nasal symptoms (Figure 1B,C).

### Effect of FA on the OVA-specific Igs and histamine release in serum

**Figure 2:**
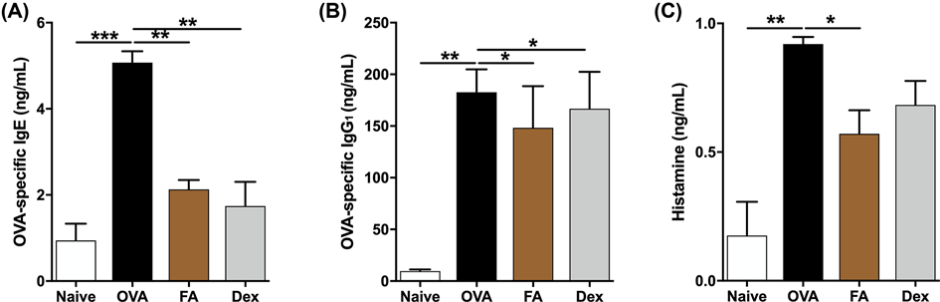
Effect of FA on levels of OVA-specific IgE, OVA-specific IgG_1_, and histamine in serum of AR mice The levels of (**A**) OVA-specific IgE, (**B**) OVA-specific IgG_1_, and (**C**) histamine in the serum were measured by ELISA kit. The values represent the mean ± S.E.M (*n*=6/group). Significant differences at ***P<0.001, **P<0.01, *P<0.05 are compared with each group.

We examined the levels of OVA-specific IgE, as well as OVA-specific IgG_1_ and histamine in the serum to determine FA’s effect on allergic responses. These levels were all significantly higher in the OVA group than in the Naive group, but levels were notably decreased after treatment with FA or Dex (Figure 2A–C).

### Effect of FA on nasal cavity inflammation in NALF and the lung tissue of AR mice

**Figure 3:**
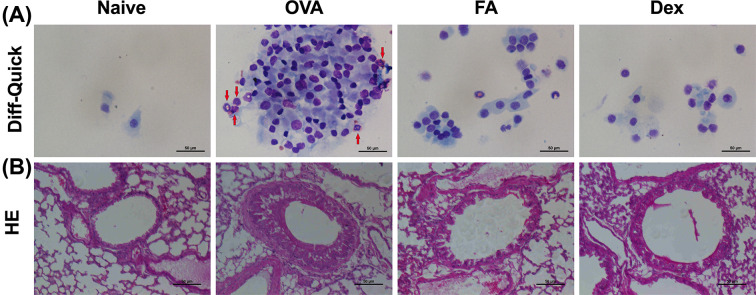
Effect of FA on nasal cavity and lung tissue of AR mice (**A**) The inflammatory cells’ infiltration into NALF. (**B**) Lung histology. The NALF were isolated by cytospin and stained with Diff-Quick. An increase in the number of eosinophils (indicated by the red arrows) in the NALF of OVA group was observed. Lung histology of AR mice showed several inflammatory features: epithelial swelling, over-secreted mucus, and severely infiltrated inflammatory cell surround bronchia. FA treatment considerably alleviated these inflammatory features. Scale bar = 50 μm.

To examine FAs effect in the AR mice model, NALF was collected after killing. The total cells number in the NALF had significantly increased in the OVA group, particularly the eosinophils, while no increase was observative the Naive group. In contrast, a markedly decreased number of cells was observed in the FA treatment group and Dex group (Figure 3A).

H&E staining revealed an inflammatory feature in the lung tissues of mice in the OVA group, in which edema and thickened bronchial epithelial cells were observed. The groups treated with FA or Dex, however, had lung tissue morphology similar to that observed in the Naive group (Figure 3B).

### Effect of FA on nasal mucosa tissue of AR mice

**Figure 4:**
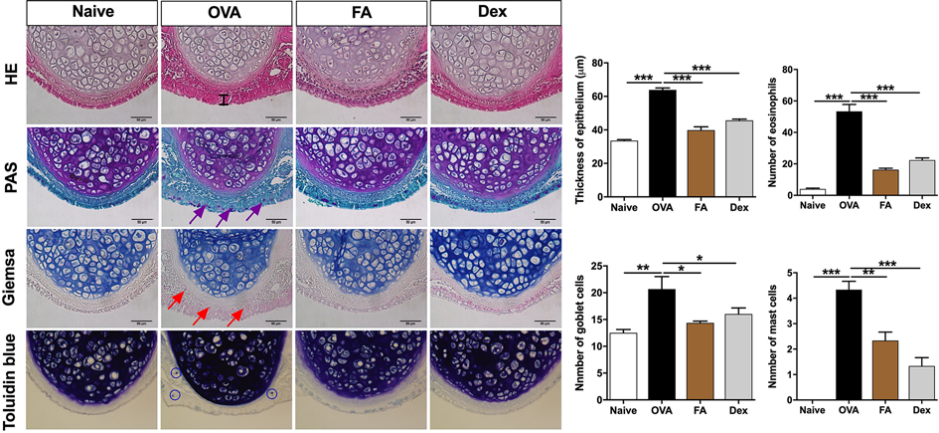
Effect of FA on nasal thickness, goblet cell hyperplasia, and eosinophil infiltration in AR nasal mucosa tissues H&E staining results, the epithelia of the OVA group were significantly thicker than those of the Naive group; epithelial swelling was ameliorated by FA and Dex treatment. The number of goblet cells (purple arrows; PAS staining), the number of eosinophils (red arrows; Giemsa staining), and the number of mast cell (blue circles; Toluidine Blue) present in the OVA group was significantly higher than in the Naive group and was markedly decreased by FA and Dex treatment. The values represent the mean ± S.E.M (*n*=6/group). Significant differences at ***P<0.001, **P<0.01, *P<0.05 are compared with each group. Bar = 50 μm.

H&E staining was used to assess histopathological changes in the nasal mucosa. In the OVA group, nasal mucosa was significantly thicker than in the Naive group. Appreciably decreased thickening of the nasal mucosa was observed in the FA and Dex treatment groups (Figure 4).

We performed PAS staining to investigate goblet cells hyperplasia in the nasal epithelium. Compared with the Naive group, considerably more goblet cells were observed in the OVA group. Subsequent to FA and Dex treatment, however, the number of goblet cells was dramatically reduced. The purple arrows indicated goblet cells (Figure 4).

We performed Giemsa staining to determine the number of eosinophils in the subepithelium. The number of eosinophils was markedly higher in the OVA group than in the Naive group. Consistent with prior results, treatment with FA and Dex resulted in a significant decrease in the number of eosinophils present. The red arrows indicated eosinophils (Figure 4).

Toluidine Blue staining reveals the presence of mast cells by highlighting them in purple. Mast cells were highly recruited in the nasal tissues of AR mice, more so than in the Naive group. After FA treatment, however, the number of infiltrated mast cells in the subepithelial and epithelial layer had markedly declined. The blue circled indicated mast cells (Figure 4).

### Effect of FA on the cytokine release in NALF of AR mice

**Figure 5:**
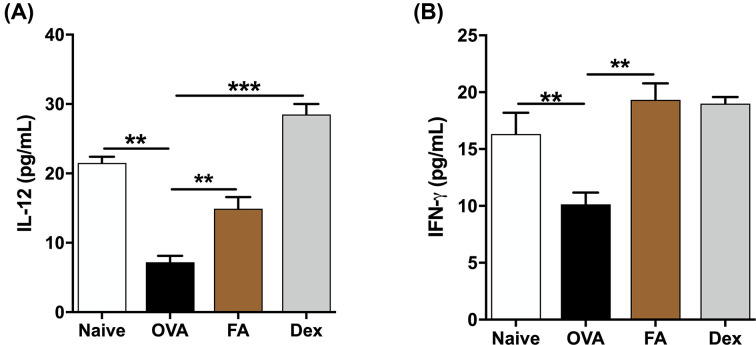
Effect of FA on the Th1 cytokines release in NALF The level of (**A**) IL-12 and (**B**) IFN-γ in NALF were determined by ELISA kit. The values represent the mean ± S.E.M (*n*=6/group). Significant differences at ***P<0.001, **P<0.01 are compared with each group.

**Figure 6:**
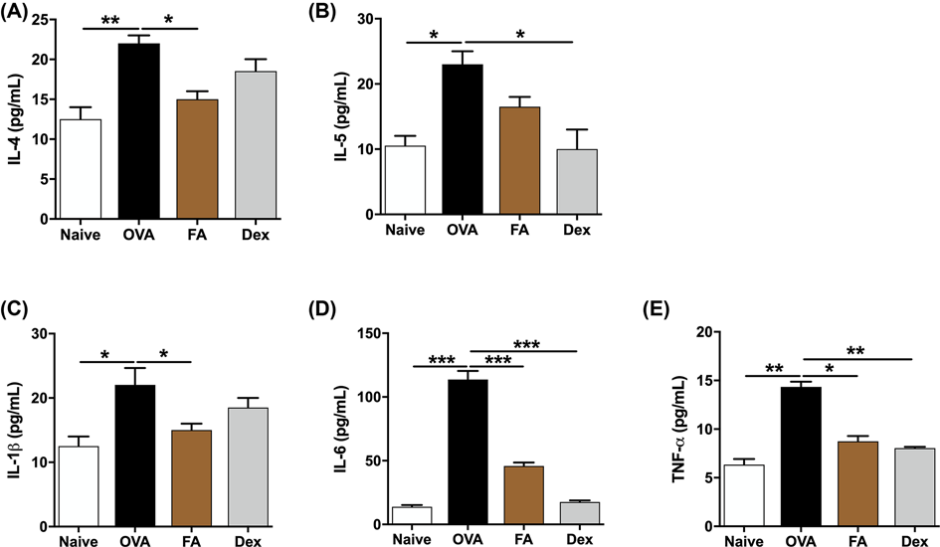
Effect of FA on the Th2 cytokines and pro-inflammatory cytokines release in NALF of AR mice The levels of (**A**) IL-4, (**B**) IL-5, (**C**) IL-1β, (**D**) IL-6, (**E**) TNF-α in NALF were determined by ELISA kit. The values represent the mean ± S.E.M (*n*=6/group). Significant differences at ***P<0.001, **P<0.01, *P<0.05 are compared with each group.

To determine FA’s effect on allergic response, we determined cytokine levels in NALF. In the OVA group, the levels of IL-4 and IL-5 (Th2-related cytokines); IL-1β, IL-6, and TNF-α (pro-inflammatory cytokines) were significantly more elevated than in the Naive group. In contrast, in the FA and Dex treatment groups, levels of those were much reduced (Figure 5A–E). IL-12 and IFN-γ (Th1-related cytokines) levels were significantly lower in the OVA group than in the Naive group. After treatment with FA or Dex, the levels of these cytokines had markedly increased (Figure 6A,B).

### Effect of FA on NF-κB signaling pathway in AR mice

**Figure 7:**
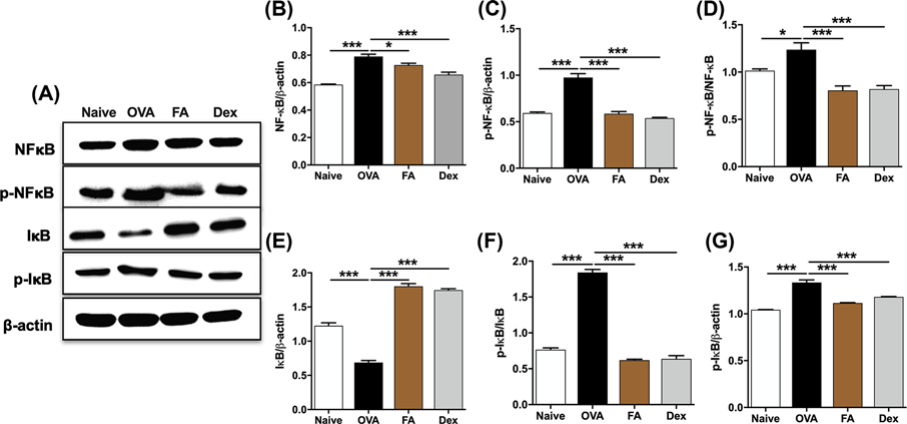
Effect of FA on NF-κB signaling pathway of AR mice (**A**) The expression of NF-κB signaling-related protein in lung tissue were quantified by Western blots. The ratios of (**B**) NF-κB/β-actin, (**C**) p-NF-κB/β-actin, (**D**) p-NF-κB/NF-κB, (**E**) I-κB/β-actin, (**F**) p-I-κB/β-actin, and (**G**) p-I-κB/I-κB were calculated via ImageJ program. Significant differences at ***P<0.001, *P<0.05 are compared with each group.

To define the underlying molecular mechanisms by which FA suppressed inflammatory response, the NF-κB signaling pathway-related proteins were quantified by Western blot. The contents of NF-κB, p-NF-κB, p-IκB in the lung tissue of the AR group were higher than Naive group, indicating that the airway tract was in an inflammation state. When FA was administered, the contents of NF-κB, p-NF-κB, p-IκB were significantly decreased, indicating FA had a significant anti-inflammatory effect on AR mouse model (Figure 7A–G; Supplementary material).

### Effect of FA on the histamine-derived mast cell release

**Figure 8:**
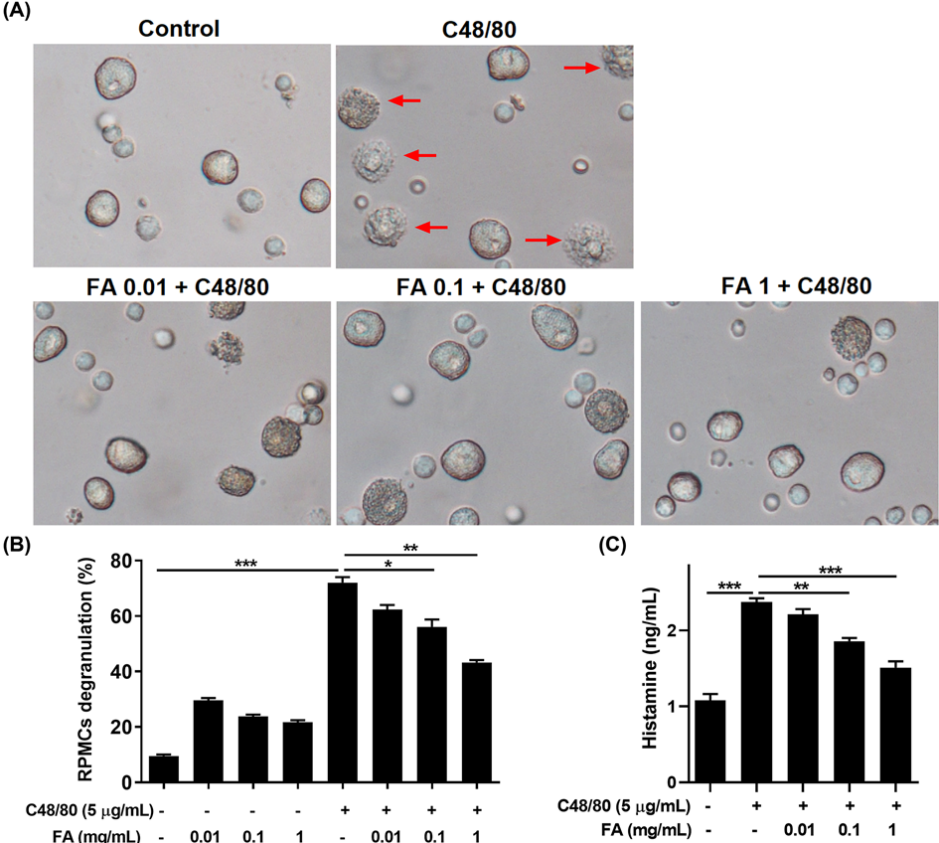
Effect of FA on the histamine derived-mast cell release (**A**) RPMC morphology, (**B**) RPMC degranulation ratio, (**C**) level of histamine release. RPMCs were pretreated with FA (0.01, 0.1, 1 mg/ml) then stimulated with compound C48/80. FA significantly alleviated RPMCs degranulation and inhibited histamine release after stimulation with compound C48/80. The red arrows indicated degranulated mast cells. Significant differences at ***P<0.001, **P<0.01, *P<0.05 are compared with each group.

RPMCs were used to investigate whether FA administration affected mast cell degranulation. After stimulation with compound C48/80, RPMCs were degranulated more rapidly compared with the non-stimulated with C48/80 RPMCs. Though pre-treated mast cells with FA 0.1 and 1 mg/ml manifested significantly less degranulation after incubation with C48/80 than the positive control group (Figure 8A,B). The red arrows indicated degranulated mast cells.

We also measured the amount of histamine-derived mast cells released after stimulation with compound C48/80. Groups pre-treated with FA presented with considerably lower levels of histamine than untreated groups. In sum, FA exerts a protective effect against RPMCs degranulation by compound C48/80 and inhibits histamine release from mast cells (Figure 8C).

## Discussion

AR is an allergic inflammation of the upper airways primarily triggered by elevated levels of serum allergen-specific IgE, penetration of inflammatory cells in the nasal mucosa, and an imbalance of Th1/Th2 cytokines [17]. IgE has been reported to mediate biological functions principally by binding to FcεRI, which is expressed by mast cells to trigger the release of mediators as a response to the presence of an allergen [18]. Histamine is a typical mast cell mediator that assumes a central role in allergic reaction chains, mediates the AR’s principal symptoms (pruritus, mucosal edema, sneezing by increased vascular permeability), and stimulates the sensory nerves to enhance secretion of mucus [19]. IgE and mast cells are involved in early-phase response to allergic inflammation, and are possibly involved in the late phase as well [20]. In the presence of mast cell or eosinophil-derived IL-4, naive T cells are differentiated towards Th2, leading to an imbalance in the Th1/Th2 ratio [21]. NF-κB also plays a role in the regulation of T-cell differentiation towards Th2 cell and produce the pro-inflammatory IL-6 and TNF-α. We investigated FA’s anti-allergic effects in an OVA-induced AR mouse model, finding that FA suppressed the clinical symptoms, IgE production, and histamine release, inactivated NF-κB signaling, and encouraged Th1/Th2 homeostasis.

Strong systemic and local allergic responses in AR mice were accompanied by a serious rise in Igs levels, particularly IgE and IgG_1_. IgE and IgG_1_, produced in a B-cell immune response, were controlled by IL-4 cytokines from Th2 cells [22]. As shown in Figure 2A,B, OVA-specific IgE and OVA-specific IgG_1_ levels were markedly up-regulated in the OVA group, more so than in the Naive group. FA treatment, however, had a statistically significant down-regulated effect on OVA-specific IgE and OVA-specific IgG_1_ secretions, suggesting the FA down-regulates the Th2 immune responses. Moreover, the elevated histamine levels present in the AR mice serum were decidedly reduced by FA or Dex treatment. RPMCs degranulation experiment confirmed the inhibition of histamine-derived mast cells release, the histamine release levels after stimulation with compound C48/80 were significantly decreased by FA pre-treatment. The mast cell degranulation ratio was also improved by FA. Based on these results, we conclude that FA manifests anti-allergic properties in both *in vivo* and *in vitro* experiments.

As the human body responds to an AR trigger, the infiltration of various inflammatory cells into nasal tissue leads to airway and nasal inflammation. Based on our investigation of histopathological changes, eosinophil infiltration, the quantity of goblet cells, and airway epithelium thickness were elevated in the OVA group, but treatment with FA ameliorated epithelial swelling in the nasal mucosa as well as in the lung tissue, and inhibited overexpression of mucus-secreting goblet cells.

**Figure 9:**
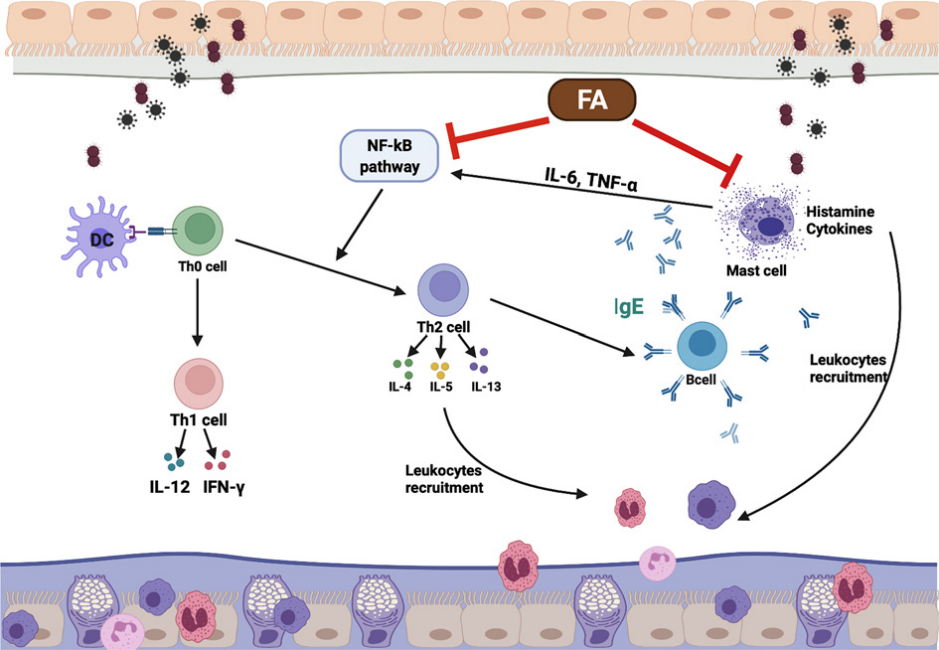
The mechanism of action FA in an OVA-induced AR mouse model Exposing to allergens promotes Th0 cell to differentiate towards Th2 cells, stimulate B cells to produce IgE, IgE bind to mast cell and activate them. Once mast cells are triggered, it releases their pro-inflammatory cytokines and chemokines. Together with Th2 cell-released cytokines, the leukocytes are recruited and activated lead to the airway allergic inflammatory symptoms. NF-κB pathway is activated to more amplify the inflammatory situation. Here, FA showed their anti-allergic inflammatory effect by balancing the Th1/Th2 ratio, suppressing the NF-κB signaling, and inhibiting mast cell degranulation.

Eosinophil-dependent inflammation and excessive activation of Th2 also play a role in triggering the symptoms of allergic inflammatory diseases [23,24]. It has been reported elsewhere that Th2 cytokines, including IL-4 and IL-5, are elevated in AR patients [25], and that these may inhibit the functions of the Th1 immune response [26]. IFN-γ is the principal Th1 effector cytokine that inhibits naive T-cell differentiation into Th2 cells and triggers macrophage production [27]. We observed that IL-4 and IL-5 levels were markedly decreased after FA treatment. In contrast, IFN-γ was higher in the FA group than in the OVA group, further substantiating FA’s ability to shift Th2 to Th1. However, in Dex group, the level of IFN-γ was not up-regulated and the level of IL-4 was not down-regulated as well. In mammals, the NF-κB family is composed of five related transcription factors: p50, p52, p65 (also RelA), c-Rel, and RelB. The transcription factors can form homo- and heterodimers, then bind to a variety of related target genes. These dimers are bound to inhibitory molecules of the IκB family of proteins (inhibitors of NF-κB) and stay under inactive state. When signaling pathway is activated, the complex NF-κB/IκB is degraded: NF-κB is phosphorylated then translocated into the nucleus and IκB is phosphorylated then degraded by IκB kinase [28]. NF-κB has been shown a critical role in the pulmonary inflammatory responses and T-cells development and functional divergence, such as Th1 and Th2 differentiation [29]. The majority role of NF-κB in T-cell differentiation is believed that related with CD4^+^ T cell. There is increasing evidence that NF-κB is the key factor of the Th0 differentiation. To be specific, mice lacking p50 or p65 are unable to mount an asthma Th2-dependent type [30]. Indeed, it fail to induce GATA3 expression lead to the decreasing Th2-related cytokines release [31]. Moreover, by binding to two major enhancer sites in the IL-4 locus, NF-κB participates in induction of IL-4 comply with nuclear factor of activated T cells lead to the differentiation of naive T cells towards Th2 cell [7]. In addition to regulate the differentiation of T naive cell, NF-κB also mediates the induction of various pro-inflammatory cytokines. The pro-inflammatory cytokines IL-1β, IL-6, and TNF-α activate NF-κB, and their expression is induced in response to NF-κB activation. Interestingly, The NFκB-related protein such as NF-κB, p-NF-κB, p-IκB were noticeably expressed in the lung tissues of AR mice compared with Naive mice; in contrast, those were obviously decreased in the FA and Dex treated mice. Therefore, by balancing the Th1/Th2 ratio, and suppressing the NF-κB signaling thereby exerting an anti-inflammatory effect, FA has the potential to help patients avoid AR symptoms (Figure 9).

Nowadays, corticosteroids and antihistamine drugs are currently the most effective medication available for the treatment of AR. However long-term use of inhaled steroids is often accompanied by undesirable adverse effects, particularly when high doses are used. FA is an effective agent and less toxicity than Dex in the AR mouse model. However, in the present study there are some limitations. First, the solubility of FA is unstable, and the other is the cross-talk between NF-κB and Th1/Th2 paradigm need to be more specified. Therefore, in the future, a p-65-deficient mice will be used for more clarification that NF-κB is the key role in the anti-allergic inflammatory effect of FA on a mouse model. Also, for delivery FA and stabling the solubility of FA by nanoparticle method will be used in the next study.

## Conclusion

We demonstrated that FA administration by oral gavage seriously attenuated airway inflammation in OVA-induced AR mouse models by suppressing inflammation cells (eosinophils, goblet cells), reducing mucus hypersecretion, regulating the balance of Th1 and Th2 cytokines, and depressing serum anti-OVA IgE, anti-OVA IgG_1_, NF-κB phosphorylation, and histamine levels. Accordingly, we conclude that FA exerts a significant anti-inflammatory effect on AR and suggest that FA may be an efficacious therapy for the treatment of AR.

## Supplementary Material

Supplementary Material

## Data Availability

The data presented in the present study are available upon request from the corresponding authors.

## Abbreviations

AR: allergic rhinitis
Dex: dexamethasone
FA: *Fructus Amomi* Cardamomi
H&E: Hematoxylin–Eosin
IFN: interferon
Ig: immunoglobulin
IL: interleukin
NALF: nasal lavage fluid
OVA: ovalbumin
PAS: Periodic Acid–Schiff
RPMC: rat peritoneal mast cell
Th: T helper

## References

[1] Bachert C., Jorissen M., Bertrand B., Khaltaev N. and Bousquet J. (2008) Allergic rhinitis and its impact on asthma update (ARIA 2008). The Belgian Perspect. 4, 253–257 19227033

[2] Sacre Hazouri J.A. (2006) [Allergic rhinitis. Coexistent diseases and complications. A review and analysis]. Rev. Alerg. Mex. 53, 9–29 16634358

[3] Galli S.J., Tsai M. and Piliponsky A.M. (2008) The development of allergic inflammation. Nature 454, 445–454 10.1038/nature07204 18650915 PMC3573758

[4] Kirmaz C., Bayrak P., Yilmaz O. and Yuksel H. (2005) Effects of glucan treatment on the Th1/Th2 balance in patients with allergic rhinitis: a double-blind placebo-controlled study. Eur. Cytokine Netw. 16, 128–134 15941684

[5] Yan L.P., Chan S.W., Chan A.S., Chen S.L., Ma X.J. and Xu H.X. (2006) Puerarin decreases serum total cholesterol and enhances thoracic aorta endothelial nitric oxide synthase expression in diet-induced hypercholesterolemic rats. Life Sci. 79, 324–330 10.1016/j.lfs.2006.01.016 16472823

[6] Sunyer J.O., Boshra H., Lorenzo G., Parra D., Freedman B. and Bosch N. (2003) Evolution of complement as an effector system in innate and adaptive immunity. Immunol. Res. 27, 549–564 10.1385/IR:27:2-3:549 12857998

[7] Liu T., Zhang L., Joo D. and Sun S.C. (2017) NF-kappaB signaling in inflammation. Signal Transduct. Target Ther. 2, 17023 10.1038/sigtrans.2017.23 29158945 PMC5661633

[8] Sheth K. (2008) Evaluating the safety of intranasal steroids in the treatment of allergic rhinitis. Allergy Asthma Clin. Immunol. 4, 125–129 10.1186/1710-1492-4-3-125 20525134 PMC2868867

[9] Mohd Zain A., Md Noh U.K., Hussein S., Che Hamzah J., Mohd Khialdin S. and Md Din N. (2019) The relationship between long-term use of intranasal corticosteroid and intraocular pressure. J. Glaucoma 28, 321–324 10.1097/IJG.0000000000001164 30585941

[10] Khattiyawittayakun L., Seresirikachorn K., Chitsuthipakorn W., Kanjanawasee D. and Snidvongs K. (2019) Effects of double-dose intranasal corticosteroid for allergic rhinitis: a systematic review and meta-analysis. Int. Forum Allergy Rhinol. 9, 72–78 10.1002/alr.22204 30179317

[11] Choi H.G., Je I.G., Kim G.J., Choi H., Kim S.H., Kim J.A. et al. (2015) Anti-allergic inflammatory activities of compounds of amomi fructus. Nat. Prod. Commun. 10, 631–632 10.1177/1934578X1501000425 25973495

[12] Yue J., Zhang S., Zheng B., Raza F., Luo Z., Li X. et al. (2021) Efficacy and mechanism of active fractions in fruit of Amomum villosum Lour. for gastric cancer. J Cancer 12, 5991–5998 10.7150/jca.61310 34539873 PMC8425199

[13] Van Nguyen T., Piao C.H., Fan Y.J., Shin D.U., Kim S.Y., Song H.J. et al. (2020) Anti-allergic rhinitis activity of alpha-lipoic acid via balancing Th17/Treg expression and enhancing Nrf2/HO-1 pathway signaling. Sci. Rep. 10, 12528 10.1038/s41598-020-69234-1 32719431 PMC7385155

[14] Piao C.H., Fan Y., Nguyen T.V., Shin H.S., Kim H.T., Song C.H. et al. (2021) PM2.5 exacerbates oxidative stress and inflammatory response through the Nrf2/NF-kappaB signaling pathway in OVA-induced allergic rhinitis mouse model. Int. J. Mol. Sci. 22, 8173 10.3390/ijms22158173 34360939 PMC8348225

[15] Bui T.T., Piao C.H., Hyeon E., Fan Y., Van Nguyen T., Jung S.Y. et al. (2019) The protective role of Piper nigrum fruit extract in an ovalbumin-induced allergic rhinitis by targeting of NFkappaBp65 and STAT3 signalings. Biomed. Pharmacother. 109, 1915–1923 10.1016/j.biopha.2018.11.073 30551446

[16] Bui T.T., Piao C.H., Song C.H., Shin H.S., Shon D.H. and Chai O.H. (2017) Piper nigrum extract ameliorated allergic inflammation through inhibiting Th2/Th17 responses and mast cells activation. Cell. Immunol. 322, 64–73 10.1016/j.cellimm.2017.10.005 29066080

[17] Shi Z., Jiang W., Wang M., Wang X., Li X., Chen X. et al. (2017) Inhibition of JAK/STAT pathway restrains TSLP-activated dendritic cells mediated inflammatory T helper type 2 cell response in allergic rhinitis. Mol. Cell. Biochem. 430, 161–169 10.1007/s11010-017-2963-7 28214951

[18] Galli S.J. and Tsai M. (2012) IgE and mast cells in allergic disease. Nat. Med. 18, 693–704 10.1038/nm.2755 22561833 PMC3597223

[19] White M.V. (1990) The role of histamine in allergic diseases. J. Allergy Clin. Immunol. 86, 599–605 10.1016/S0091-6749(05)80223-4 1699987

[20] Chai O.H. and Song C.H. (2015) Role of mast cell in the late phase of contact hypersensitivity induced by trimellitic anhydride. Anat. Cell Biol. 48, 225–234 10.5115/acb.2015.48.4.225 26770872 PMC4701695

[21] Broide D.H. (2010) Allergic rhinitis: pathophysiology. Allergy Asthma Proc. 31, 370–374 10.2500/aap.2010.31.3388 20929602

[22] Zhao N., Liu Y., Liang H. and Jiang X. (2016) Bone marrow-derived mesenchymal stem cells reduce immune reaction in a mouse model of allergic rhinitis. Am. J. Transl. Res. 8, 5628–5636 28078033 PMC5209513

[23] Martin L.B., Kita H., Leiferman K.M. and Gleich G.J. (1996) Eosinophils in allergy: role in disease, degranulation, and cytokines. Int. Arch. Allergy Immunol. 109, 207–215 10.1159/000237239 8620088

[24] Akdis C.A., Arkwright P.D., Bruggen M.C., Busse W., Gadina M., Guttman-Yassky E. et al. (2020) Type 2 immunity in the skin and lungs. Allergy 75, 1582–1605 10.1111/all.14318 32319104

[25] Sun Y.Q., Deng M.X., He J., Zeng Q.X., Wen W., Wong D.S. et al. (2012) Human pluripotent stem cell-derived mesenchymal stem cells prevent allergic airway inflammation in mice. Stem Cells 30, 2692–2699 10.1002/stem.1241 22987325 PMC3549478

[26] Peterson J.D., Herzenberg L.A., Vasquez K. and Waltenbaugh C. (1998) Glutathione levels in antigen-presenting cells modulate Th1 versus Th2 response patterns. Proc. Natl. Acad. Sci. U.S.A. 95, 3071–3076 10.1073/pnas.95.6.3071 9501217 PMC19696

[27] Kidd P. (2003) Th1/Th2 balance: the hypothesis, its limitations, and implications for health and disease. Altern. Med. Rev. 8, 223–246 12946237

[28] Hoesel B. and Schmid J.A. (2013) The complexity of NF-kappaB signaling in inflammation and cancer. Mol. Cancer 12, 86 10.1186/1476-4598-12-86 23915189 PMC3750319

[29] Gerondakis S., Fulford T.S., Messina N.L. and Grumont R.J. (2014) NF-kappaB control of T cell development. Nat. Immunol. 15, 15–25 10.1038/ni.2785 24352326

[30] Hayden M.S. and Ghosh S. (2011) NF-kappaB in immunobiology. Cell Res. 21, 223–244 10.1038/cr.2011.13 21243012 PMC3193440

[31] Das J., Chen C.H., Yang L., Cohn L., Ray P. and Ray A. (2001) A critical role for NF-kappa B in GATA3 expression and TH2 differentiation in allergic airway inflammation. Nat. Immunol. 2, 45–50 10.1038/83158 11135577

