# Correction: Fructus Amomi extract attenuates nasal inflammation by restoring Th1/Th2 balance and downregulation of NF-kB phosphorylation in OVA-induced allergic rhinitis

**DOI:** 10.1042/BSR-2021-2681_COR

**Published:** 2023-05-23

**Keywords:** allergic arthritis, Fructus Amomi, mast cell degranulation, NF-ΚB phosphorylation, Th1/Th2 balance

The authors of the original article “Fructus Amomi extract attenuates nasal inflammation by restoring Th1/Th2 balance and downregulation of NF-kBphosphorylation in OVA-induced allergic rhinitis” (*Biosci Rep*. 2022 42(3): BSR20212681 doi: 10.1042/BSR20212681) would like to correct Figure 7A and the corresponding raw blots represented in the Supplementary Figure 1D. The authors have stated that in checking the western blots from Figure 7, they found an error that occurred whilst cropping the bands, which resulted in the p-lkB bands appearing in the wrong position. The requested correction has been assessed and agreed by the Editorial Board. The authors declare that these corrections do not change the results or conclusions of their paper. The corrected versions of Figure 7 and Supplementary Figure 1 are presented here.

**Figure 7:**
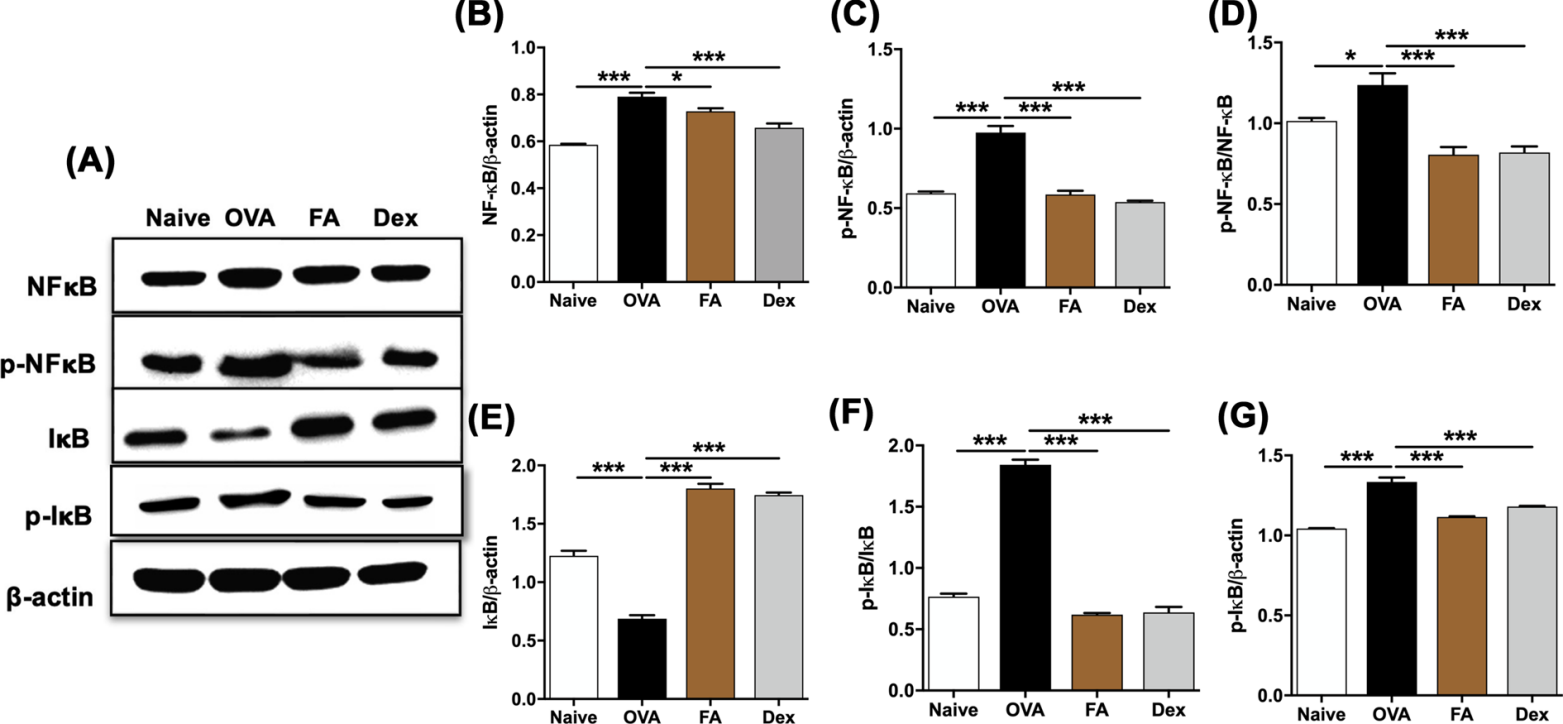
Effect of FA on NF-κB signaling pathway of AR mice **(A)** The expression of NF-κB signaling-related protein in lung tissue were quantified by Western blots. The ratios of **(B)** NF-κB/β-actin, **(C)** p-NF-κB/β-actin, **(D)** p-NF-κB/NF-κB, **(E)** I-κB/β-actin, **(F)** p-I-κB/β-actin, and **(G)** p-I-κB/I-κB were calculated via ImageJ program. Significant differences at ***P<0.001, *P<0.05 are compared with each group.

## Supplementary Material

Supplementary Figure 1

