# Supplementary material for: Correction: Fructus Amomi extract attenuates nasal inflammation by restoring Th1/Th2 balance and downregulation of NF-kB phosphorylation in OVA-induced allergic rhinitis
Source: Biosci Rep. 2023 May 23;43(5):BSR-2021-2681_COR. doi: 10.1042/BSR-2021-2681_COR (PMC10214086; doi:10.1042/BSR-2021-2681_COR)
Supplement: Supplementary Figure 1 [file BSR-2021-2681_COR_supp.pdf]

Supplementary material for

Fructus Amomi extract attenuates nasal inflammation by restoring Th1/Th2 balance via the downregulation of NF-κB phosphorylation and mast cell activation in OVA-induced allergic rhinitis

Yanjing Fan[a,†], Thi Van Nguyen[a,†], Chun Hua Piao[a,b], Hee Soon Shin[c,d], Chang Ho Song[a,e*], Ok Hee Chai[a,e*]

[a]Department of Anatomy, Jeonbuk National University Medical School, Jeonju, Jeonbuk, 54896, Republic of Korea.

[b]Department of Pulmonary and Critical Care Medicine, Yantai Yuhuangding Hospital, Yantai 264000, China.

[c]Division of Food Functionality Research, Korea Food Research Institute, 245 Nongsaengmyeong-ro, Iseo-myeon, Wanju-gun, Jeollabuk-do, 55365, Republic of Korea.

[d]Food Biotechnology Program, Korea University of Science and Technology, Daejeon, 305-350, Republic of Korea.

[e]Institute for Medical Sciences, Jeonbuk National University, Jeonju, Jeonbuk, 54896, Republic of Korea.

† These authors equally contributed in this work.

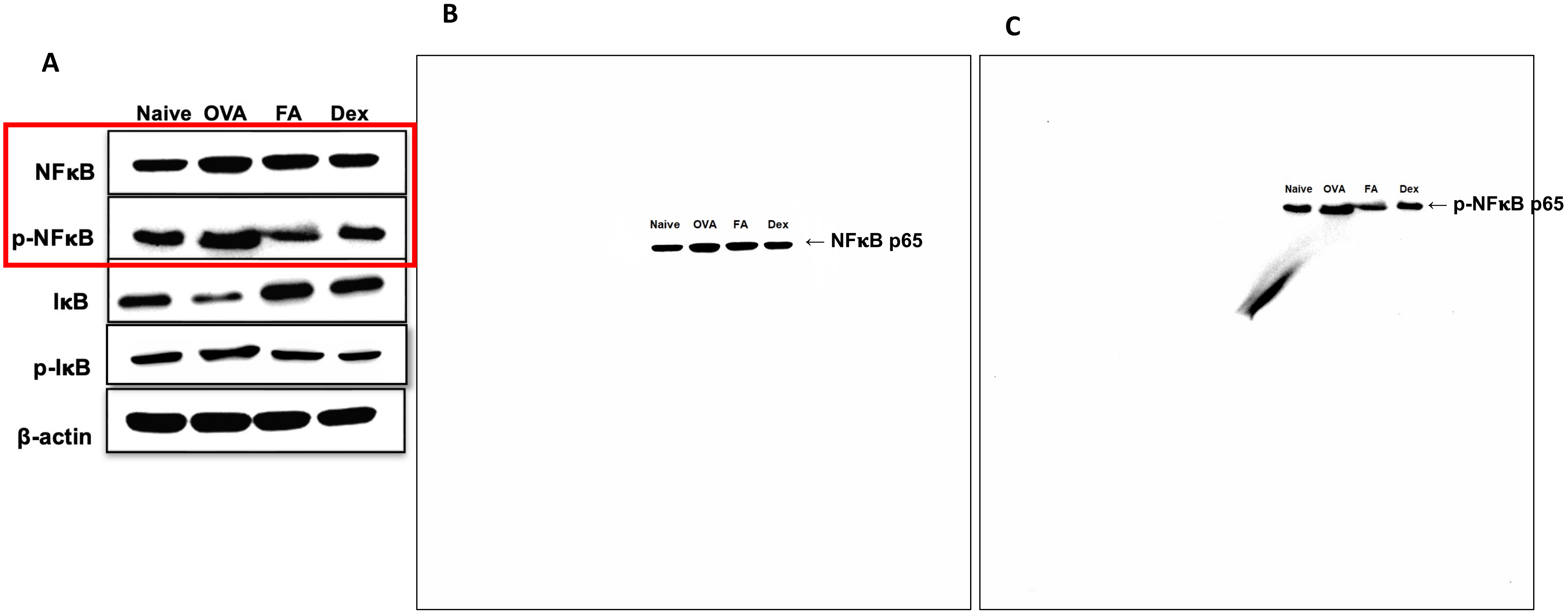

Figure S1. **Full length gels and blots of NFκB signaling related proteins in lung tissues**
(A) Western blot data. Orginal Western blot picture of (B) NF**κ**B p65 and (C) p-NF**κ**B p65. Membrane C was stripped from membrane B.

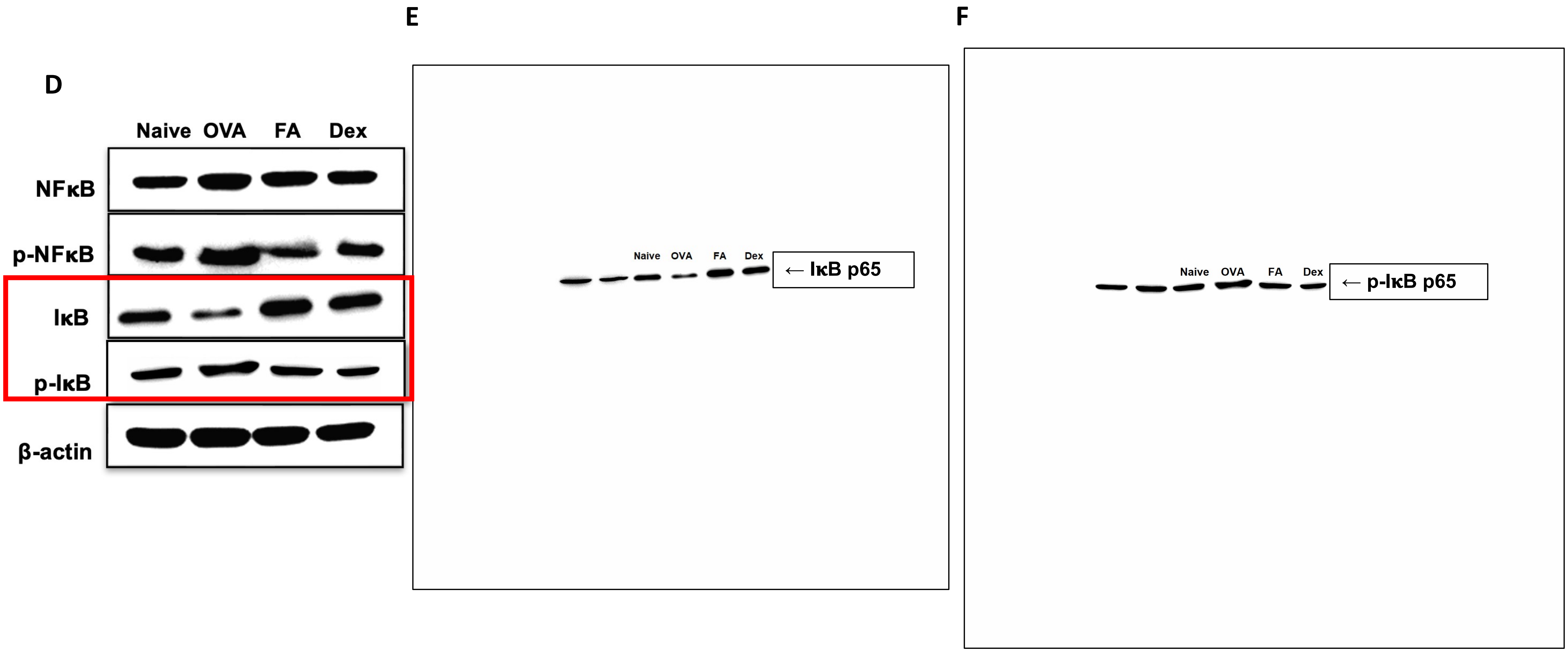

Figure S1. Full length gels and blots of NFκB signaling related proteins in lung tissues
(D) Western blot data. Orginal Western blot picture of (E) IκB and (C) p-IκB. Membrane E was stripped from membrane F.

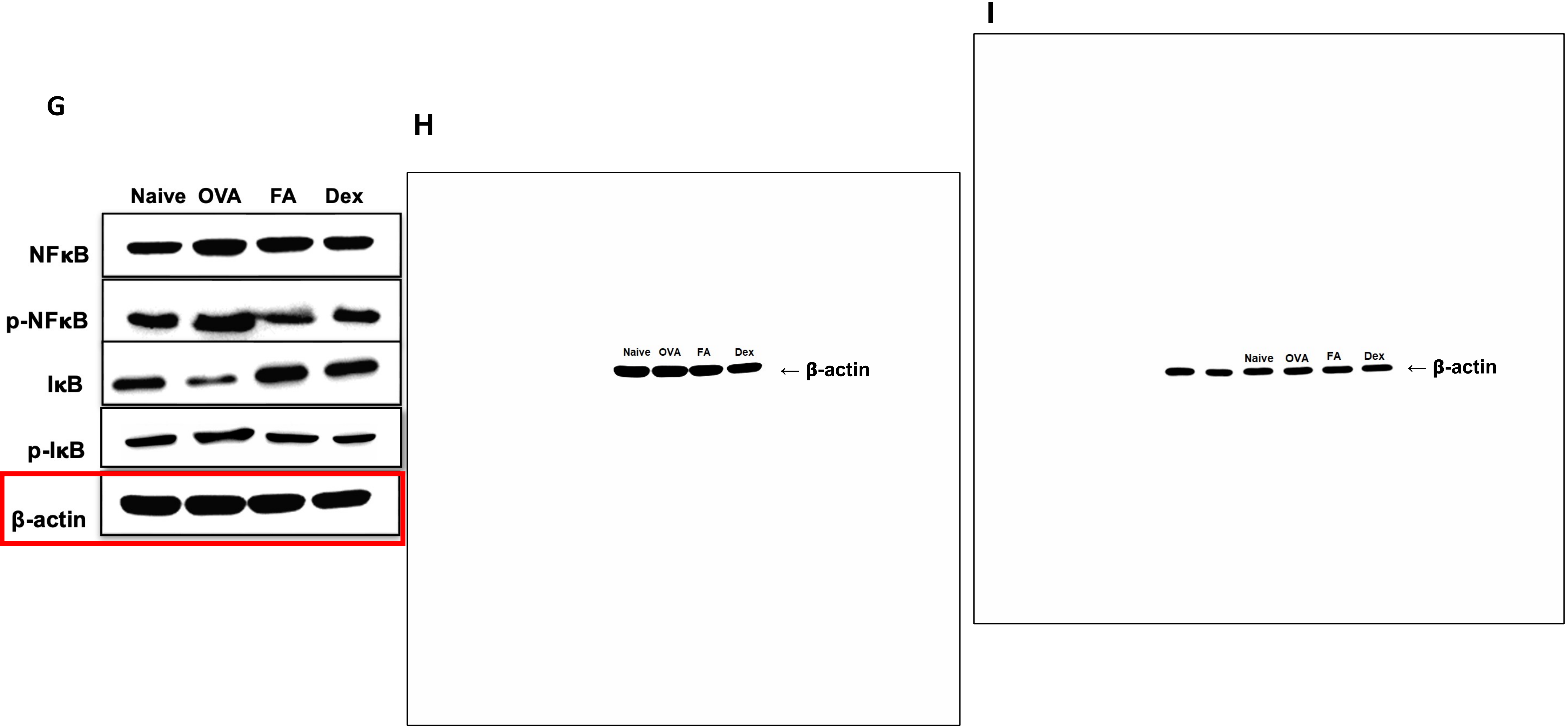

Figure S1. Full length gels and blots of NFκB signaling related proteins in lung tissues
(A) Western blot data. Orginal Western blot picture of (H), (I) **β**-actin. Membrane H was stripped from membrane A. Membrane I was stripped from membrane F.

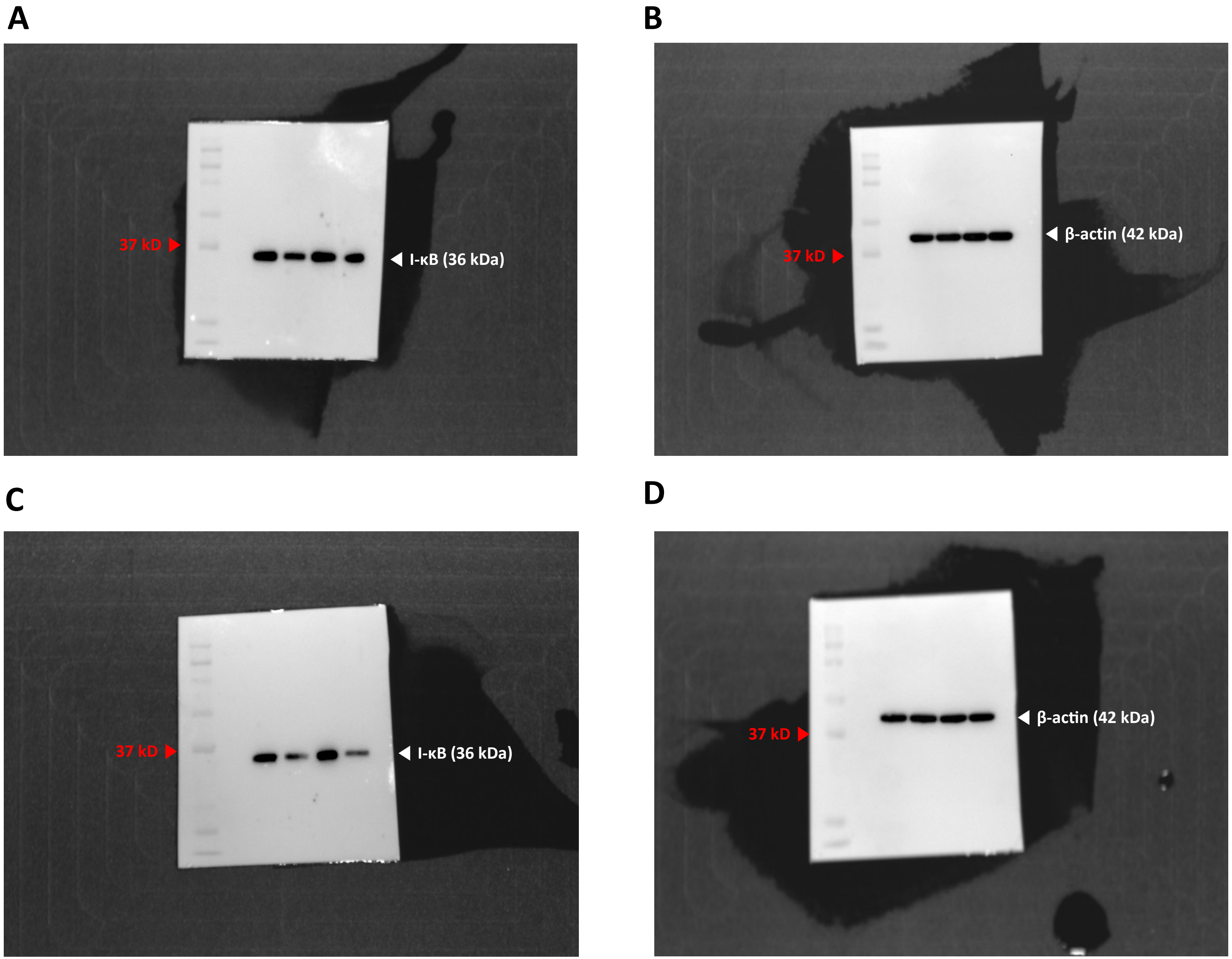

Figure S2. Full length gels and blots of I-κB proteins in lung tissues (repeated experiment) Orginal Western blot picture of (A), (C) I-κB; (B), (D) **β**-actin. Membrane B was stripped from membrane A. Membrane D was stripped from membrane C.

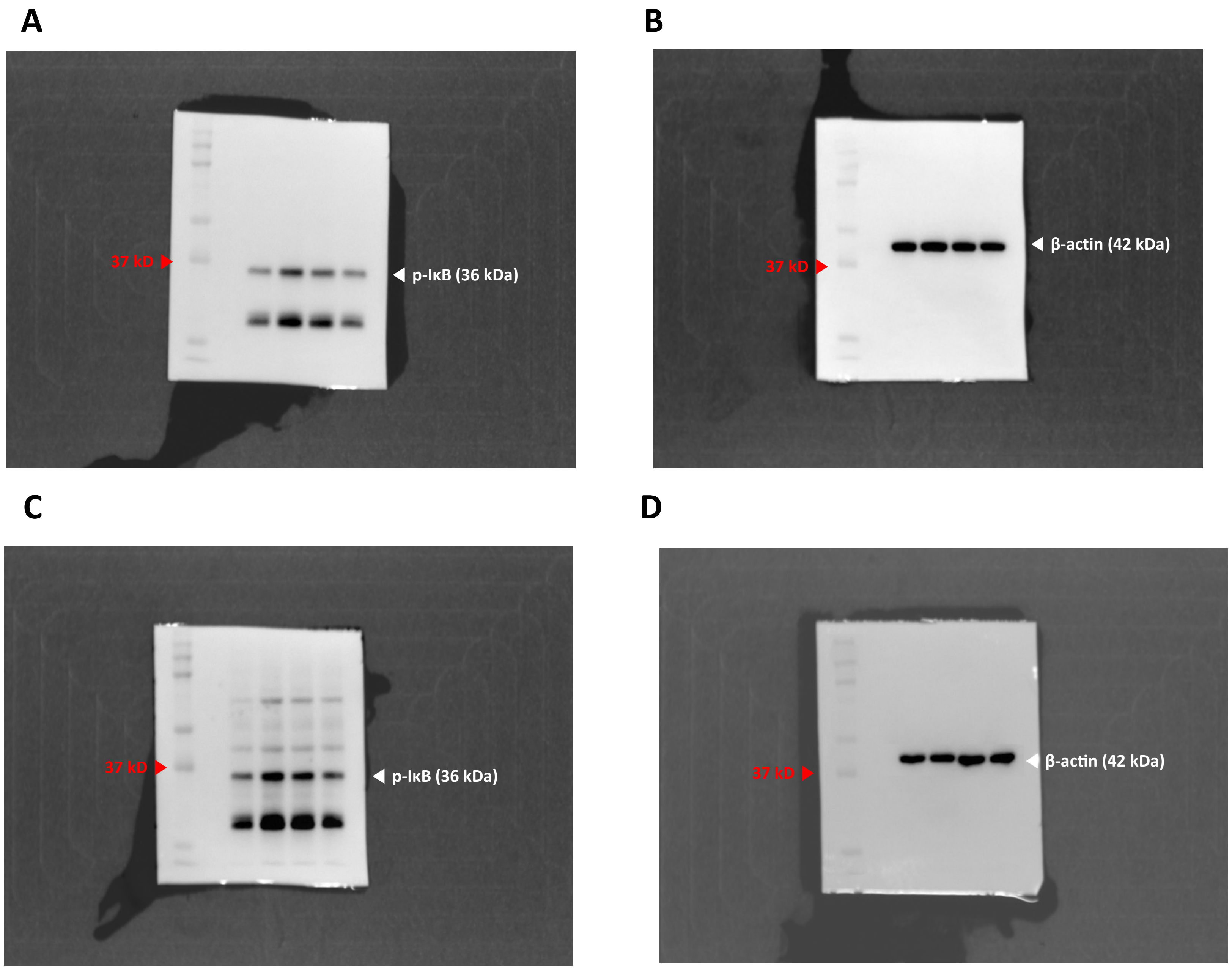

Figure S3. Full length gels and blots of p-IκB proteins in lung tissues (repeated experiment) Orginal Western blot picture of (A), (C) p-IκB; (B), (D) **β**-actin. Membrane B was stripped from membrane A. Membrane D was stripped from membrane C.

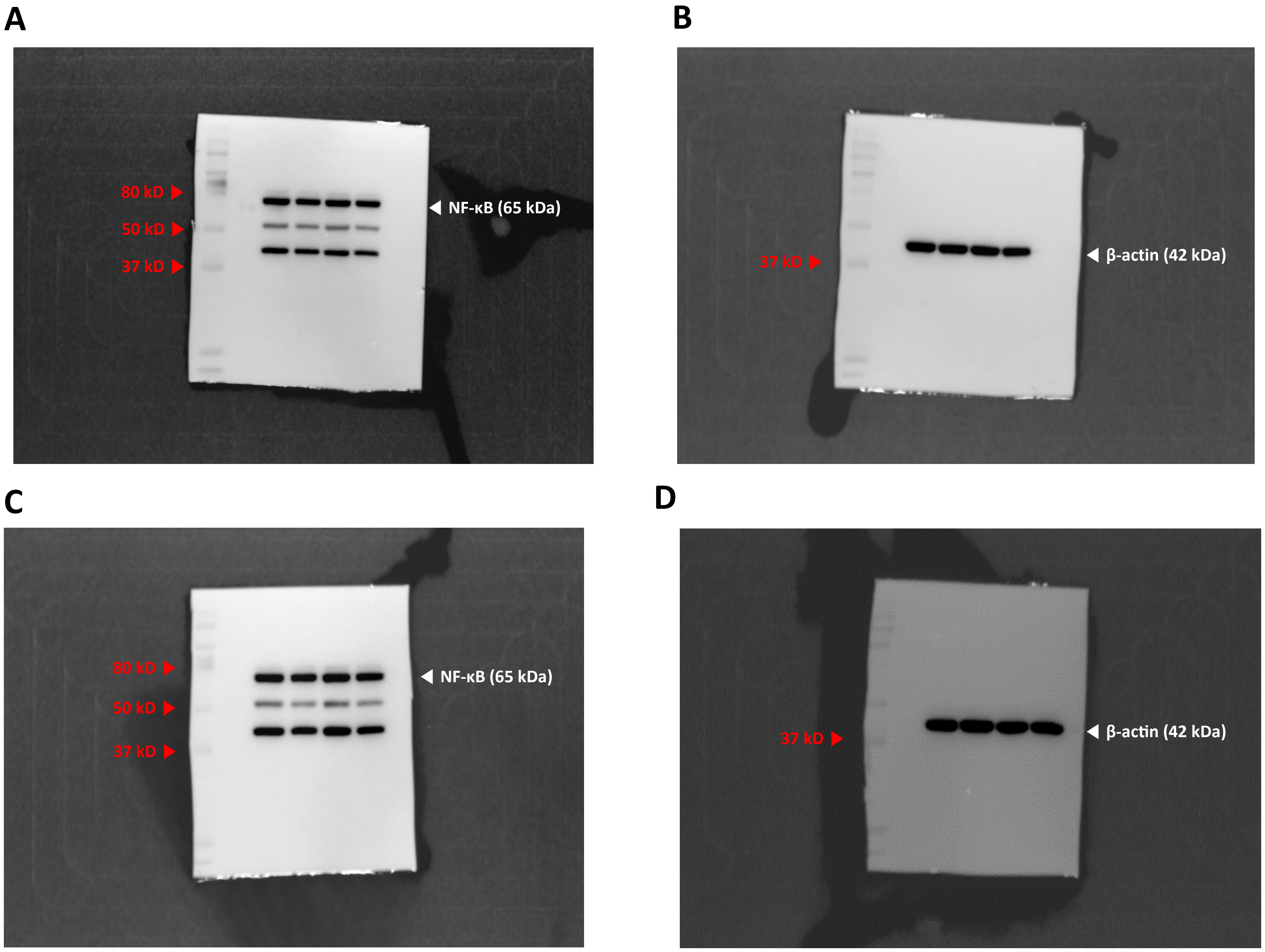

Figure S4. **Full length gels and blots of NF-κB proteins in lung tissues (repeated experiment)** Orginal Western blot picture of (A), (C) NF-κB; (B), (D) **β**-actin. Membrane B was stripped from membrane A. Membrane D was stripped from membrane C.

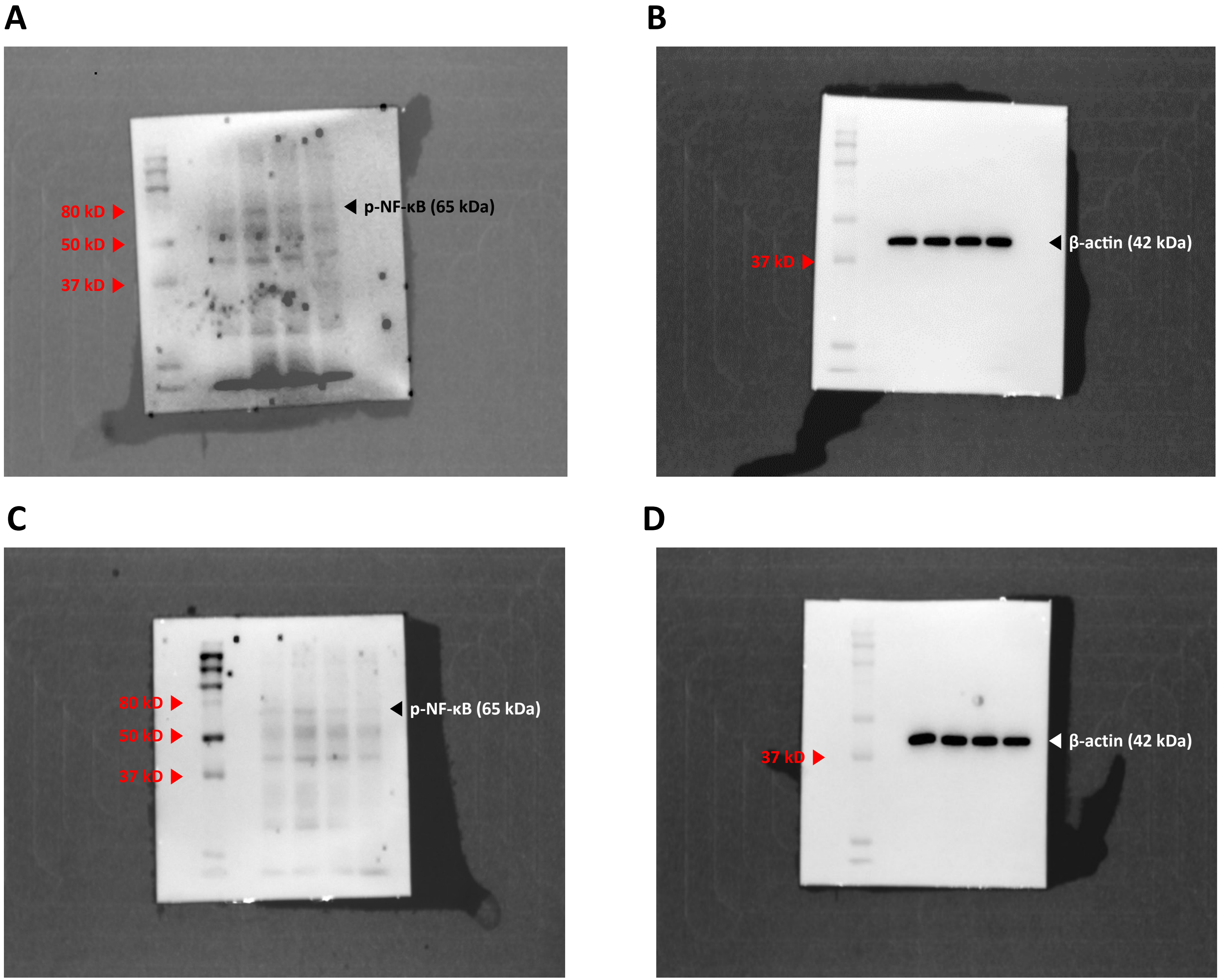

Figure S5. Full length gels and blots of p-NF-κB proteins in lung tissues (repeated experiment) Orginal Western blot picture of (A), (C) p-NF-κB; (B), (D) **β**-actin. Membrane B was stripped from membrane A. Membrane D was stripped from membrane C.
